# The Sigma 2 receptor promotes and the Sigma 1 receptor inhibits mu-opioid receptor-mediated antinociception

**DOI:** 10.1186/s13041-020-00676-4

**Published:** 2020-11-11

**Authors:** Pilar Sánchez-Blázquez, Elsa Cortés-Montero, María Rodríguez-Muñoz, Manuel Merlos, Javier Garzón-Niño

**Affiliations:** 1 Neuropharmacology, Cajal Institute, Consejo Superior de Investigaciones Científicas (CSIC), Doctor Arce 37, 28002 Madrid, Spain; 2 Drug Discovery & Preclinical Development, Esteve, Barcelona, Spain

**Keywords:** Sigma 2 receptor, Sigma 1 receptor, Knockout mice, Mu opioid receptor, Neuropathic pain, Analgesia

## Abstract

The Sigma-1 receptor (σ1R) has emerged as an interesting pharmacological target because it inhibits analgesia mediated by mu-opioid receptors (MOR), and also facilitates the development of neuropathic pain. Based on these findings, the recent cloning of the Sigma-2 receptor (σ2R) led us to investigate its potential role as a regulator of opioid analgesia and of pain hypersensitivity in σ2R knockout mice. In contrast to σ1R deficient mice, σ2R knockout mice developed mechanical allodynia following establishment of chronic constriction injury-induced neuropathic pain, which was alleviated by the σ1R antagonist S1RA. The analgesic effects of morphine, [D-Ala, *N*-MePhe, Gly-ol]-encephalin (DAMGO) and β-endorphin increased in σ1R^−/−^ mice and diminished in σ2R^−/−^ mice. The analgesic effect of morphine was increased in σ2R^−/−^ mice by treatment with S1RA. However, σ2R^−/−^ mice and wild-type mice exhibited comparable antinociceptive responses to the delta receptor agonist [D-Pen2,5]-encephalin (DPDPE), the cannabinoid type 1 receptor agonist WIN55,212-2 and the α2-adrenergic receptor agonist clonidine. Therefore, while σR1 inhibits and σ2R facilitates MOR-mediated analgesia these receptors exchange their roles when regulating neuropathic pain perception. Our study may help identify new pharmacological targets for diminishing pain perception and improving opioid detoxification therapies.

## Introduction

Sigma receptors (σRs) are unique transmembrane proteins expressed throughout the central nervous system and in certain peripheral tissues. Based on current classifications, there are two types of these receptors, namely, the sigma-1 receptor (σ1R) and the sigma-2 receptor (σ2R) [1–4]. The σ1R was initially identified in 1976 as a member of the plasma membrane opioid receptor family [5], while σ2R was not discovered until later. For many years, σRs were described to bind to radioligands in preparations of brain synaptosomes. [^3^H]( +)-pentazocine exhibits a high affinity for σ1R, whereas [^3^H]DTG binds with equal affinity to both σ1R and σ2R. Subsequent studies have revealed that these proteins are also involved in intracellular ion regulation and neuron survival [1, 4, 6–8].

The σ1R was purified, sequenced and cloned from guinea pig brain in 1996, and it bears little sequence homology to any known mammalian receptor [9]. On the other hand, it has been postulated that σ2R complexes with progesterone receptor membrane component 1 (PGRMC1). The recent molecular cloning of σ2R identified this protein as the TMEM97 protein [10–12]. Some evidence suggest that σ2R is also involved in cholesterol trafficking and homeostasis [13] and in the regulation of intracellular calcium levels [14]. Notably, σ2R is involved in several disease states, and the utility of its exogenous ligands as cancer therapeutics and diagnostic tools has been reported [15–17]. Additionally, σ2R has been implicated in multiple neurodegenerative and neurological disorders [18, 19]. Similar to the ligands of σ1R, certain molecules that bind to σ2R also reduce mechanical hypersensitivity in a spared nerve injury model [20].

The availability of *σ2R*^−/−^ (σ2R knockout) mice, deficient in TMEM97/σ2R, have allowed us to investigate the potential role of this receptor in pain sensitivity. Because σ1R participate in a tonic anti-opioid system [21, 22], we also evaluated the capacity of σ2R to modulate opioid induced analgesia. We observed that σ2R-deficient mice do not exhibit overt physical or behavioral abnormalities. Most importantly, we found that σ2R contributes to the analgesic effects of MOR agonists but not those of delta opioid or cannabinoid type 1 receptor agonists.

## Materials and methods

### Animals and drugs

Male albino CD-1 mice (ENVIGO, Barcelona, Spain), wild-type (WT) mice, σ2R^−/−^ (allele name Tmem97tm1.1(KOMP)Vlcg) and σ1R^−/−^ mice were used in the study. The genetically modified σ2R^−/−^ mice were on the C57BL/6NTac background and were originally purchased from UC Davis KOMP Repository (MMRRC Stock #: 050148-UCD, Davis, CA, USA). σ1R^−/−^ mice were backcrossed (N10 generation) onto a CD1 albino genetic background were obtained from (ENVIGO, Milano, Italy). The mice used in these experiments were produced from heterozygous breeding pairs and assigned randomly to be used for the different experiments. The σ2R^−/−^ mice exhibited no noticeable differences from their WT littermates with respect to appearance, body size, or morphologic parameters. The genotypes of the WT and σ2R^−/−^ mice were confirmed by PCR. Each DNA sample was amplified using two sets of primers and a PCR thermocycler (Eppendorf Iberica SLU, Madrid, Spain). One set of primers consisted of Reg-Tmem97-wtF (AGAGTAAAGGGCTAGCCAGGAAACC) and Reg-Tmem97-wtR (GGTGTCACACACCTTTAATCCCAGC). This set was responsible for amplifying the WT sequence (320 bp). The second set consisted of Reg-LacF (ACTTGCTTTAAAAAACCTCCCACA) and Reg-Tmem97-R (TCCTTCCCTGTAACCCATTTCTGGC). This set of PCR primers was responsible for amplifying the deleted sequence (722 bp). Each DNA sample was run with both sets of primers (Sigma‐Aldrich, Madrid, Spain) to determine whether the mice were WT, σ2R^−/−^ or heterozygous. The PCR thermal cycling protocol included two steps. The first step was as follows: denaturation at 94 °C for 15 s followed by 65 °C for 30 s and then 72 °C for 40 s. This series was repeated for 10 cycles, with the second temperature decreasing by 1 °C each cycle. Directly following the first step, the second step was performed as follows: denaturation at 94 °C for 15 s followed by 55 °C for 30 s and 72 °C for 40 s. The second step was repeated for 30 cycles, and a final elongation at 72 °C for 5 min was performed.

All mouse housing, breeding and experimental protocols were performed in strict accordance with the guidelines of the European Community for the Care and Use of Laboratory Animals (Council Directive 2010/63/EU) and Spanish law (RD53/2013) regulating animal research. The use of drugs, experimental design and sample size determination were approved by the Ethical Committee for Research of the CSIC (SAF2015–65420 & CAM PROEX 225/14). The mice were maintained at 22 °C on a diurnal 12-h light/dark cycle and provided free access to food and water. Male mice were specifically selected to avoid the potentially confounding variable of the female estrus cycle. To reduce the risk of social stress, mice from the same litter were grouped together and remained in these groups throughout the study. The mice were also provided extra space for comfort, as well as nesting material (e.g., soft paper and cardboard refuge) and small pieces of chewable wood. The experiments were performed in different cohorts of mice to avoid any variations caused by handling stress. The mice were used when they were between the ages of 6 and 10 weeks. All attempts were made to minimize the number of mice used in each experiment.

### Behavioral outcomes

Before behavioral testing began, we allowed the mice to familiarize themselves with the testing room for two consecutive days (60 min/day). On the day of testing, we transferred the mice to the testing room 30 min prior to the test session. To prevent potential changes in behavior, we performed each test on a different cohort of animals. Initial screening included body weight and contact-righting reflex measurements.

*Exploratory behavior*. This test was performed in a 14 × 14 inch arena with a lattice containig 16 holes in the floor (Cibertec, Madrid, Spain). The arena was fitted with photocells to count the number of hole pokes during each 10 min trial. In addition, rearing, center activity, and peripheral activity were also recorded. A variation in exploratory behavior was defined as a change in the number of hole pokes without a change in the other activities.

#### Spontaneous activity

The mice were tested individually using 20 cm × 20 cm × 28 cm transparent plastic automated activity monitors (Accuscan activity analyzer -Versamax 260 v2.4; Omnitech Electronics, Inc., OH, USA). Infrared beam crossings were recorded for 100 min at 10 min intervals. At the end of each session, the mice were returned to their home cages, and the boxes were wiped clean with a 10% alcohol solution.

#### Rota-Rod

Motor coordination was measured using an accelerated rotarod (Ugo Basile). Each animal was trained to use the rotarod at a constant acceleration over six 5 min sessions with an interval of 20 min between trials. On the following days, the mice were again tested, and the time to fall from the rod was measured with a cutoff time of 5 min.

#### Passive avoidance task

The acquisition and retention of passive avoidance behaviors were examined using identical illuminated and non-illuminated (20 cm^3^ × 10 cm^3^ × 15 cm^3^) boxes separated by a guillotine door (5 cm^2^ × 5 cm^2^) as previously described [23]. Each mice participated in two separate trials. First, in the acquisition trial, each mouse was initially placed in the light compartment, and the door between the two compartments was opened after 10 s. When the mouse entered the dark compartment, the guillotine door automatically closed, and an electrical foot shock (0.5 mA, 3 s) was delivered through the floor. The latency time to enter the dark chamber was recorded. Only mice that entered the dark chamber within 60 s were subjected to a retention trial. For the retention trial, each mouse was again placed in the light compartment, and the latency to enter the dark compartment was recorded (up to 10 min).

### Nerve injury pain model

After the basal mechanical sensitivity of the mice was tested, neuropathic pain was induced by chronic sciatic nerve constriction injury (CCI) surgery under isoflurane/oxygen anesthesia [24] using the procedure described by Bennett and Xie [25] a modifications. Briefly, a 0.5 cm incision was made in the right midthigh, the biceps femoris muscle was separated, and the sciatic nerve was exposed proximal to its trifurcation. Two ligatures (5/0 braided silk suture; Lorca Marin, Murcia, Spain, 70,014) were tied around this nerve approximately 1 mm apart until a short flick of the ipsilateral hind limb was observed. The incision was then closed in layers with a 4–0 Ethicon silk suture. The same procedure was used for sham surgery except that the sciatic nerve was exposed but not ligated. The tactile pain threshold of both the ipsilateral and contralateral hind paws were then assessed on days 0, 3, 7, and 12 post-surgery. The mice were individually placed in a transparent plastic cage with a wire mesh bottom that allowed full access to the paws. After a habituation period of 20 min, a mechanical stimulus was delivered to the plantar surface from below the floor of the test chamber to measure allodynia using an automatic von Frey apparatus (Ugo Basile #37,450, Comerio, Italy). A steel rod (0.5 mm diameter) was pushed against the hind paw over a 10 s period as the force increased from 0 to 10 g. When the mouse withdrew its hind paw, the mechanical stimulus was automatically stopped, and the force at which withdrawal occurred was recorded. At each time point, three separate threshold measurements were obtained from each hind paw and then averaged.

### Evaluation of antinociception and acute tolerance

The response of the animals to nociceptive stimuli was determined by the warm water (52 °C) tail-flick test as previously described [22, 26]. In this tail-flick analgesic test, a thermal noxious stimulus is applied to promote flicking of the mouse’s tail, and opioids given intracerebroventricularly (icv) increase the time elapsed between application of the stimulus and the flick. This response involves a spinal reflex that is facilitated by the brain stem nociceptive modulating network. Baseline latencies ranged from 1.6 to 2.1 s. A cut-off time of 10 s was used to minimize the risk of tissue damage. Drugs were icv injected into the lateral ventricles in a volume of 4 μL, and antinociception was assessed at different time intervals thereafter. Saline was likewise administered as a control. Antinociception is expressed as a percentage of the maximum possible effect (MPE = 100 × [test latency-baseline latency]/[cut-off time (10 s)-baseline latency]).

The development of morphine acute tolerance was monitored when a priming dose of 10 nmol (WT mice) or 30 nmol (σ2R^−/−^ mice) had no effect on baseline latencies. Thus, 24 h later, a test dose of morphine was injected icv and analgesia was measured at the post-injection interval of 30 min.

The compounds used were morphine sulfate (Merck, Darmstadt, Germany); β-endorphin (GenScript, USA); DAMGO (#1171, Tocris); DPDPE (#1431, Tocris); WIN55,212–2 (#1038, Tocris); clonidine (#0690, Tocris). S1RA: 4-[2-[[5-methyl-1-(2-naphthalenyl)-1H-pyrazol-3-yl]oxy]ethyl] morpholine), was obtained from Esteve Pharmaceuticals (Barcelona, Spain). To facilitate selective and direct access to their targets, the compounds were each injected into the lateral ventricles of mice in a volume of 4 μL volume as previously described [22, 26]. The animals were lightly anesthetized, and the drugs were injected icv 2 mm lateral and 2 mm caudal from bregma, and at a depth of 3 mm with a 10 μL Hamilton syringe. The drugs were infused at a rate of 1 μL every 5 s. After that, the needle was maintained for an additional 10 s. Eight to 10 mice were treated with each compound. Test drugs were dissolved in saline, and the doses and treatment intervals were selected based on previous studies and pilot assays. The motor performance of mice administered the solvents used was identical to non-injected animals.

In a series of experiments, the expression of σ2R was reduced by subchronic administration of synthetic end-capped phosphorothioate antisense oligodeoxy-nucleotides (Sigma-Aldrich, Spain, USA). The ODN σ2R was 5′ A*C*GACTGGCAAGCCGGTGAT*A*G 3′ (adapted from [27]). A random ODN (ODN RD) served as a control [26, 28]. The animals were injected with either the vehicle, ODN RD or antisense oligodeoxynucleotide into the right lateral ventricle over a 5 day period. On day 6, the analgesic compounds were injected icv, and the antinociceptive activity evaluated.

### Reverse-transcription (RT)-PCR

Total RNA was isolated by using TRIzol Reagent (Invitrogen, USA) and first-strand cDNA was prepared from total RNA with an oligo(dT) 18 primer and AMV reverse transcriptase (BioFlux, Japan) according to the manufacturer's instructions. The primers used for subsequent PCR were, σ2R: 5′-GCGTGCGATCGCCGGGGCCCTGGCAGCTAGGC-3′ (forward) and 5′-TTGTGTTTAAACTTTTTTCTTTCTTTTCTCCTCATACTTGT-3 (reverse); σ1R: 5´-ATTGGCGATCGCCCCGTGGGCCGCGGGACGG-3´ (forward) and 5´-ATTAGTTTAAACGGAGTCTTGGCCAAAGAGGTAG-3´(reverse); HINT1: 5´-GGCTGCGATCGCCGCTGACGAGATTGCCAAG-3´ (forward) and 5´- GTCGGTTTAAACACCAGGAGGCCAGTTCATCT-3´ (reverse); MOR: 5´-AGGAGCGATCGCCGCTGTATTTATTGTCTGCTGGACC-3´ (forward) and 5´-GCGAGTTTAAACGGGCAATGGAGCAGTTTCTGCTT-3´ (reverse); GAPDH: 5´-CATCACCATCTTCCAGGAGC-3´ (forward) and 5´-ATCACAAACATGGGGGCATCG-3´ (reverse). The PCR products were electrophoresed on 2% agarose gel, stained with ethidium bromide, and visualized under UV illumination. The intensities of the specific bands were analyzed and quantified.

### Statistical analysis

Graphs were constructed and statistical analyses were performed using Sigmaplot v.14 (SPSS Science Software). The data were analyzed using 2-way ANOVA with genotype and treatment as main factors. A significant interaction was detected for all experiments, and the follow-up analysis involved 1-way ANOVAs for each genotype and treatment followed by all pairwise Holm-Sidak multiple comparison tests, as indicated in the figure legends. Statistical significance was defined as *p* < 0.05.

## Results

### ***Characterization of σ2R***^−/−^*** mice***

**Fig. 1:**
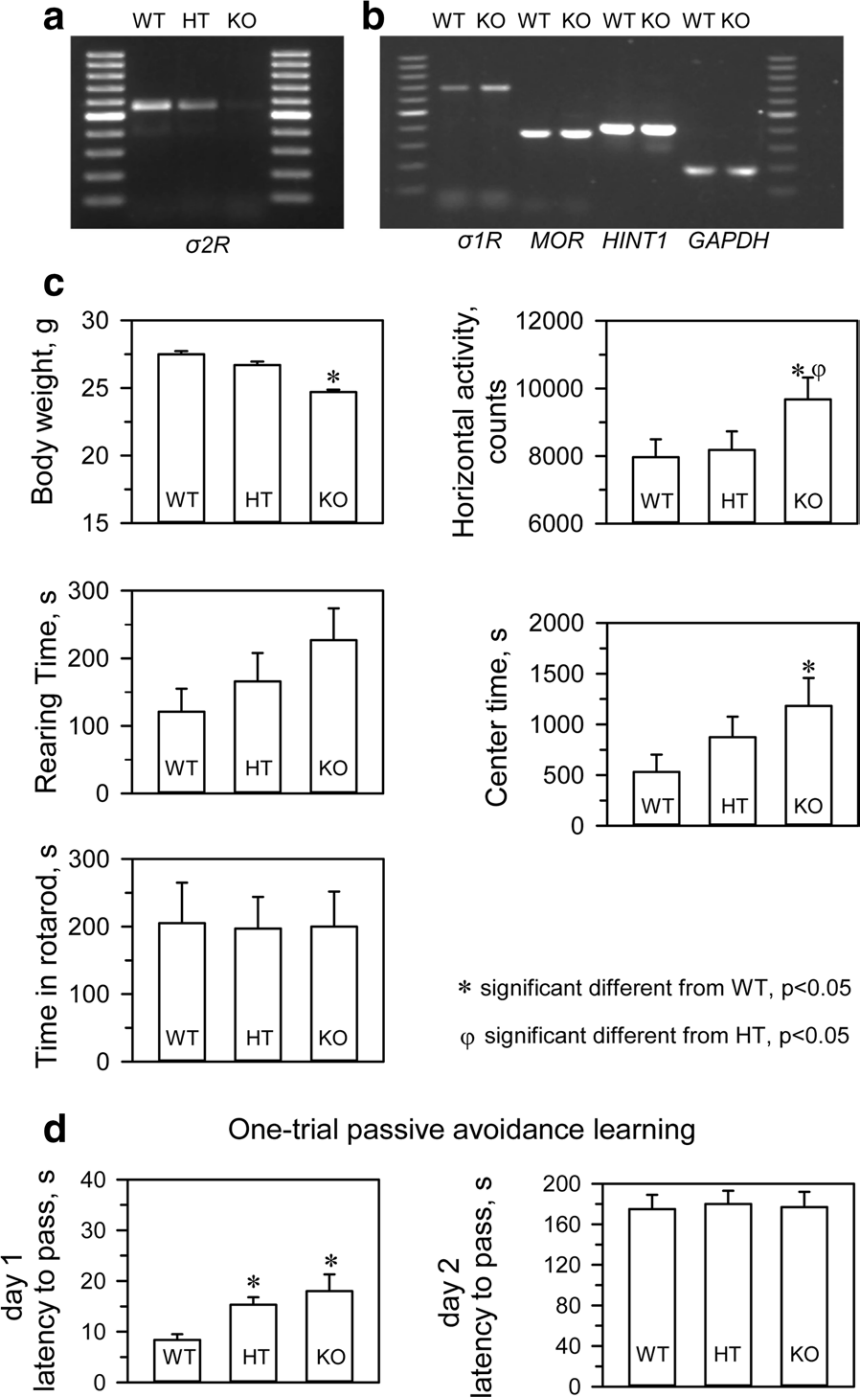
Analysis of mRNA levels and phenotypic evaluation of transgenic mice. **a** PCR analysis of a litter from a single mating showing wild-type (WT) (+ / +), heterozygous (HT) (+ / −), and homozygous (−/−) σ2R mice. **b** Deletion of *σ2R* did not significantly alter the expression of *σ1R*, *HINT1* or *MOR*. **c** Body weight gain in grams, horizontal activity, rearing time in seconds, and latency to fall from the rotarod in seconds for the three groups of mice. **d** Learning performance in the passive avoidance test. Six to eight mice (5 weeks of age) were subjected to each treatment, and the data represent the means ± SEMs. * Indicates significantly different from the WT mice, degrees of freedom (df) = 16, all data were analyzed by pairwise Holm-Sidak multiple comparison tests following ANOVA, *p* < 0.05. σ1R, HINT1 and MOR represents sigma type 1 receptor, histidine triad nucleotide-binding protein 1, and mu-opioid receptor, respectively

We confirmed that σ2R^−/−^ mice (KOMP Repository, MMRRC Stock #: 050148-UCD, Davis, CA, USA) did not express *σ2R* mRNA in brain tissue (Fig. 1a). Targeted deletion of the *σ2R* gene was not accompanied by compensatory changes in the levels of mRNAs encoding critical proteins in our study, such as σ1R, MOR or MOR- and σ1R-regulated histidine triad nucleotide-binding protein 1 (HINT1) (Fig. 1b). σ2R-deficient mice bred normally and did not present evident physical or behavioral abnormalities at birth. At weaning (3 to 6 weeks old), σ2R^−/−^ mice were smaller than WT mice (*p* < 0.05). However, by week 8, the differences in body weight were no longer significant. The locomotor performance of the mice was then evaluated by analyzing three basic parameters: horizontal activity, time spent in the center area, and rearing. While σ2R^−/−^mice exhibited a similar exploratory behavior and rearing activity as control, they exhibited increased activity and spent more time in the center area (Fig. 1c). The motor coordination of both groups of mice was comparable when evaluated with an accelerating rotarod.

The WT and σ2R^−/−^ mice were also subjected to an inhibitory avoidance paradigm that tests cognition/memory. A retention trial was conducted 24 h after the training trial. No significant differences were observed between WT and σ2R^−/−^ mice in the retention trial. It should be noted that both groups did show an increase in latency in the retention trial compared to the training trial, which was interpreted as learning (Fig. 1d).

### ***Chronic constriction injury in WT and σ2R***^−/−^*** mice***

**Fig. 2:**
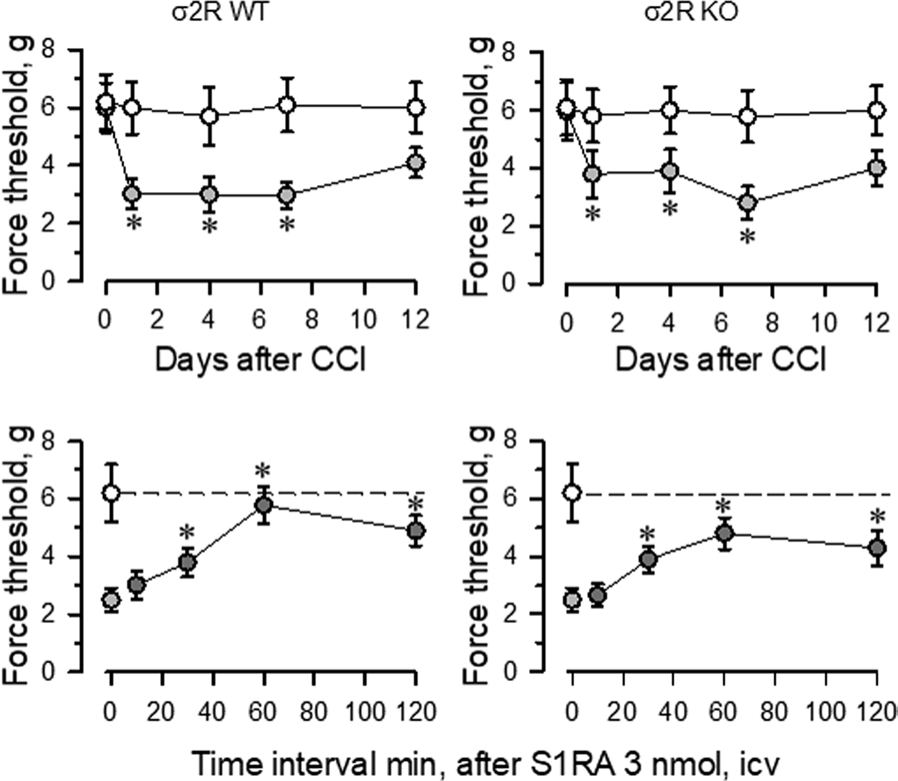
Induction of mechanical allodynia in WT and σ2R^−/−^ mice. Chronic constriction injure (CCI) of the sciatic nerve caused neuropathic pain in mice. The paw withdrawal thresholds of the contralateral and ipsilateral paw of the wild-type (WT; left panel) and knockout (KO; right panel) mice were measured before (indicated as 0) and 1, 4, 7, and 12 days after surgery. The force (in grams) at which the mice withdrew their paws in response to von Frey hair stimulation was determined as an index of mechanical allodynia. All data are presented as the mean ± SEM of six mice. * Indicates significantly different compared to the nociceptive threshold of the sham-operated control group on day 0 (7th after surgery); *p* < 0.05. Lower panels: the effect of the σ1R antagonist S1RA on the mechanical allodynia displayed by WT and σ2R^−/−^ mice. Antiallodynic compound was administered icv 7 days after surgery, and the nociceptive threshold was evaluated at the indicated post-injection intervals (in minutes). The dashed line indicates the typical nociceptive threshold obtained of the contralateral paw. * Indicates significantly different compared to the ipsilateral paw; all data were analyzed by pairwise Holm-Sidak multiple comparison tests following ANOVA; *p* < 0.05

Mice with CCI-induced neuropathic pain display a series of behavioral and molecular changes that are diminished upon treatment with antiallodynic substances such as σ1R antagonists [29]. Thus, we assessed the possible relevance of σ2R in the development of neuropathic pain. Nerve-injured WT and σ2R^−/−^ mice maintained a healthy appearance and were well groomed. The body weights of both groups of mice decreased after surgery but returned to preoperative values within 2–4 days. Before surgery (day 0), WT and σ2R^−/−^ mice displayed similar responses to the mechanical nociceptive stimulus (Fig. 2). Seven days after surgery, sham-operated and CCI-exposed σ2R^−/−^ mice displayed similar responses of the contralateral paw as WT animals. On the other hand, from 1 to 7 days after surgery both groups of CCI mice showed identical levels of allodynia in the ipsilateral nerve-injured legs, and on days 12 to 15, the nociceptive responses of both groups of CCI mice returned to presurgery levels. Icv administered S1RA (E-52862), a highly selective σ1R antagonist [30], reduced the allodynia induced by the CCI model in WT and σ2R^−/−^ mice. The peak antiallodynic effect was observed 60 min after the administration of S1RA (Fig. 2).

### Influence of σ2R on the antinociceptive response to morphine

**Fig. 3:**
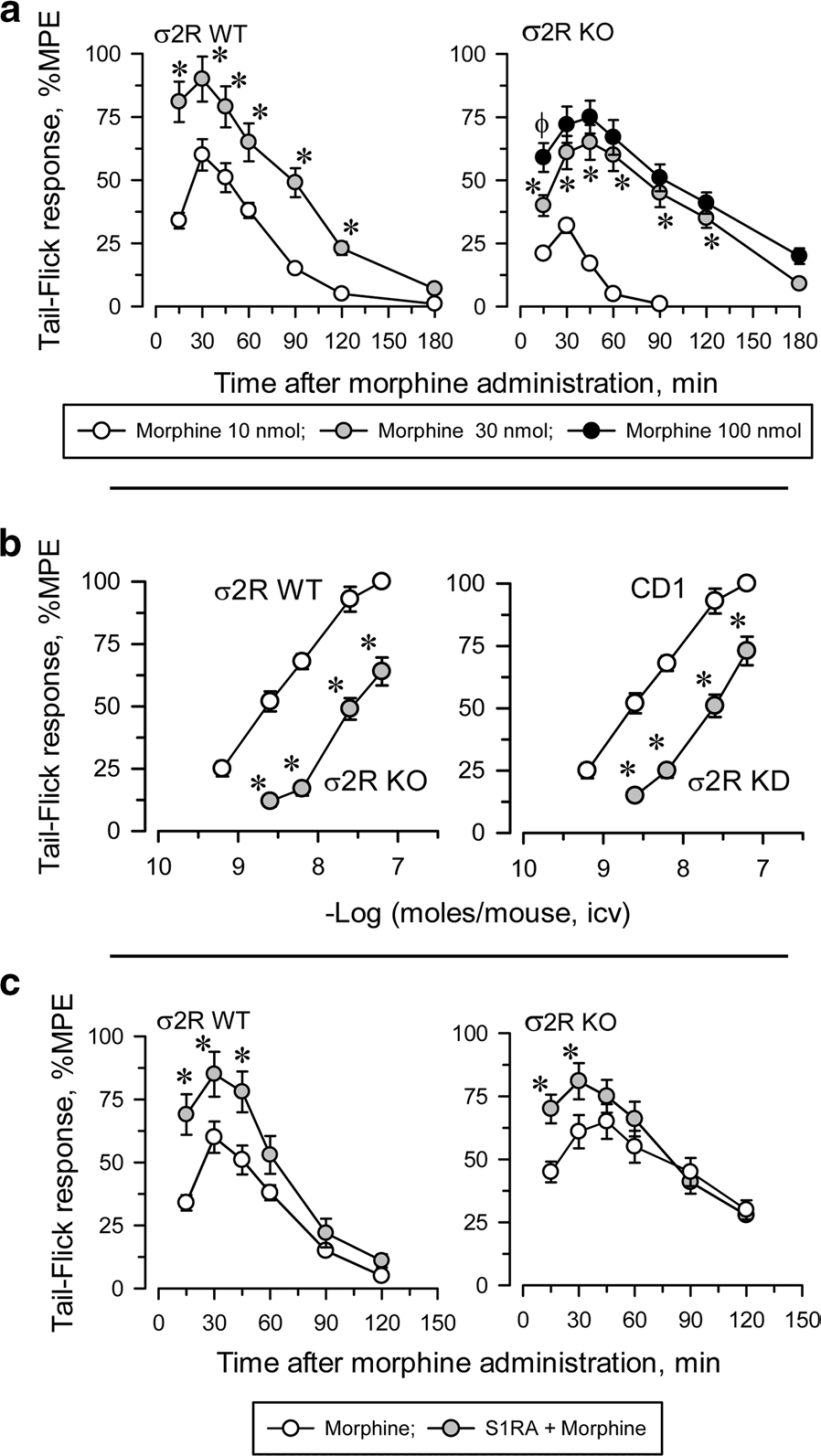
σ2R promotes morphine-induced supraspinal analgesia. **a** Wild-type (WT) and σ2R^−/−^ (KO) mice were icv injected with increasing doses of morphine, and antinociception was monitored at different intervals by the warm water (52 °C) tail-flick test. Each point is the mean ± SEM of groups of six mice. For every post-opioid interval, * indicates a significant difference compared to the group that received 10 nmol morphine. **b** Dose response curves of morphine in WT, σ2R^−/−^ mice (left panel) and of antisense oligonucleotide-induced σ2R knockdown (KD) CD1 mice and controls treated with a mismatched oligodeoxinucleotide (RD-M; right panel). The analgesic effect was evaluated at point of the peak effect, i.e., 30 min after morphine injection. Each point is the mean ± SEM of groups of six mice. * Indicates a significant difference compared to the WT (RD-M) group. **c** Mice were icv injected with 3 nmol S1RA 20 min before treatment with 10 nmol (WT) or 30 nmol (KO) morphine, and analgesia was evaluated 30 min later. The points are the mean ± SEM of the data from six mice. For every postopioid interval, * indicates that S1RA produced a significantly different response than morphine only. All data were analyzed by pairwise Holm-Sidak multiple comparison tests following ANOVA; *p* < 0.05

Icv administered morphine produces a dose-dependent antinociceptive effect when evaluated by the thermal tail-flick test (Fig. 3). In WT mice, the antinociceptive effect peaked approximately 30 min after injection and decreased after 120 min. The effect of morphine in σ2R^−/−^ mice was significantly lower than in WT animals (Fig. 3a). The apparent ED50 of icv-administered morphine was 4.84 nmol (95% confidence interval: 3.63–6.43) for control mice and 22.10 nmol (19.34–24.72) for σ2R^−/−^ mice (Fig. 3b). Basal latencies were not different between σ2R^−/−^ mice and WT mice (1.61 ± 0.14 and 1.74 ± 0.13, respectively; n = 10).

Antisense oligodeoxynucleotides are useful tools for reducing neural protein expression, and their selectivity in terms of related signaling proteins has been described elsewhere [26, 31]. We observed that the response of σ2R^−/−^ mice and σ2R knockdown mice to morphine were identically decreased (Fig. 3b). It is known that in naïve mice, the administration of S1RA increases morphine antinociception [22, 30]. The ED70 of icv morphine in our analgesic paradigm was 10 nmol in WT mice and 30 nmol in σ2R^−/−^ mice. Icv administered S1RA (3 nmol) increased the analgesic activity of morphine in both groups of mice (Fig. 3c).

**Fig. 4:**
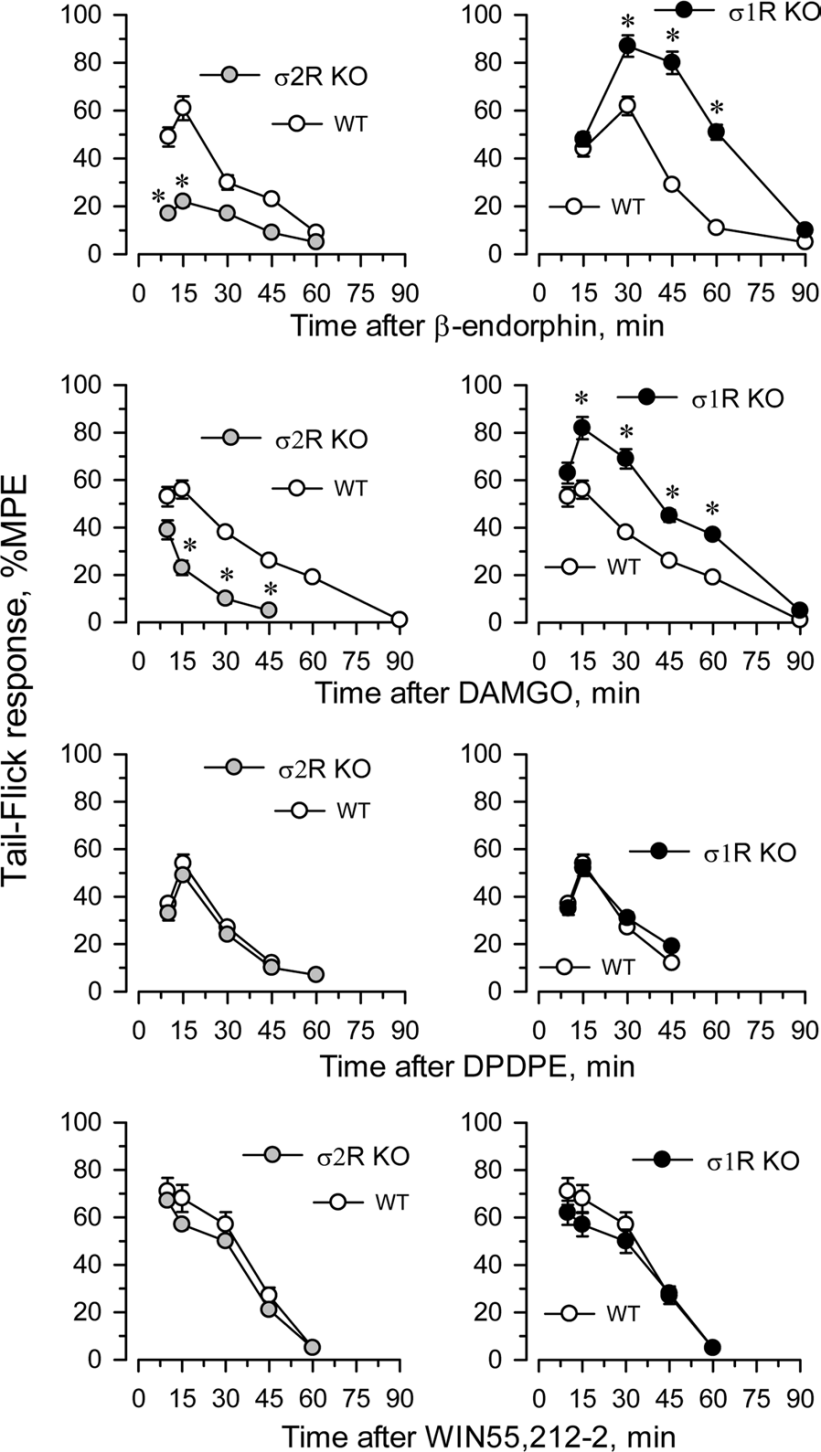
Effect of σ1R or σ2R deletion on analgesia induced by opioids and nonopioid compounds. Analgesic compounds were icv injected, and the time course of analgesia was evaluated in σ2R^−/−^ (KO; left panel), σ1R^−/−^ mice (KO; right panel) and corresponding wild-type mice (WT). Analgesia was determined by the warm water (52 °C) tail-flick test at the indicated intervals postinjection. The values are mean ± SEM of groups of 6–8 mice. * Indicates significantly different compared to WT mice; all data were analyzed by pairwise Holm-Sidak multiple comparison tests following ANOVA; *p* < 0.05

The influence of targeted deletion of *σ1R* gene on MOR-induced antinociception is a known issue [22]. While the antinociceptive effects of DAMGO and β-endorphin were diminished in σ2R^−/−^ mice, they were increased in σ1R^−/−^ mice (Fig. 4). The ability of these σ receptors to regulate analgesia promoted by activation of G receptors other than MOR was explored. The deletion of either form of σ receptor did not alter the analgesic activity of representative agonists of other G-receptors implicated in analgesia, such as the delta opioid receptor (DOR) agonist DPDPE, the cannabinoid receptor type 1 (CB1R) agonist WIN55,212-2 (Fig. 4) and the α2-adrenergic receptor (α2AR) agonist clonidine (not shown).

**Fig. 5:**
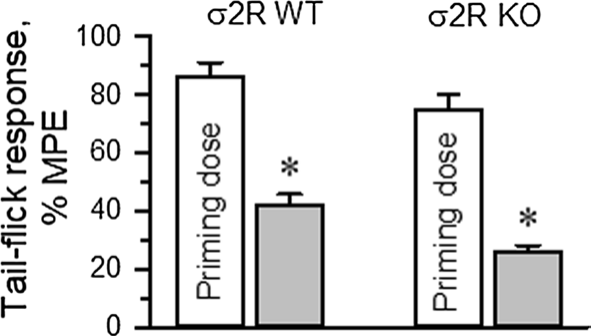
Development of single-dose morphine-induced tolerance. The σ2R^−/−^ (KO) mice developed a profound and lasting tolerance to acute administration of morphine (10 nmol, icv). The data are expressed as the mean ± SEM. * Indicates significantly different compared to the effects induced by the morphine priming dose (30 min) in wild-type (WT) and σ2R^−/−^ mice; all data were analyzed by pairwise Holm-Sidak multiple comparison tests following ANOVA; *p* < 0.05

The influence of σ2R on the production of opioid-induced acute tolerance was also investigated. Mice received either saline (control) or morphine, and 24 h later, the analgesia evoked by a second injection of morphine was evaluated. Since mice showed a low analgesic response to morphine, to obtain comparable analgesic effects in both experimental groups, the dose of morphine administered to the σ2R^−/−^ mice was increased to promote approximately 80% of the maximum possible effect (MPE) in our paradigm. A priming dose of morphine was icv injected into WT (10 nmol) and σ2R^−/−^ mice (30 nmol), and the effect of their respective morphine ED80s was evaluated 24 h later. In WT mice, the analgesic effect of the ED80 decreased from 86 ± 5% MPE to 42 ± 4% MPE 24 h after the priming dose of 10 nmol morphine. Deletion of σ2R did not prevent the development of acute tolerance, and ED80 antinociception dropped from about 75 ± 5% to 26 ± 4% MPE (Fig. 5).

## Discussion

Because there are currently no reliable antibodies (with sensitivity and selectivity) testing the σ2R protein in neuronal tissue, PCR was used to confirm the absence of *σ2R* mRNA in the knockout animals provided by UC Davis KOMP Repository. σ2R^−/−^ mice showed exploratory behavior, locomotor performance, motor coordination and cognitive abilities comparable to those of WT mice. Furthermore, like naïve WT mice, naïve σ2R^−/−^ mice responded to a wide range of mechanical stimulus intensities (from innocuous to noxious). Consequently, targeted deletion of the *σ2R* gene did not affect normal mechanical stimulus perception or the motor response necessary to produce paw withdrawal. Nerve-injured WT and σ2R^−/−^ mice subjected to CCI showed similar levels of allodynia on day 7 after surgery. Then, the absence of the σ2R receptor did not lead to significant alterations in the pathogenesis of neuropathic pain.

Several studies have demonstrated that σ1R^−/−^ mice do not develop allodynia in different animal models of neuropathic pain such as CCI [33], paclitaxel [34], spinal cord contusion injury [35], or spare nerve injury [36]. Accordingly, σ1R antagonists reduce nerve injury-induced mechanical hypersensitivity in WT mice [30, 32]. Consistent with this idea, we observed that administration of the selective σ1R antagonist S1RA reduced allodynia in WT and σ2R^−/−^ mice. On the other hand, molecules that bind to σ2R/Tmem97 as putative agonists reduce mechanical hypersensitivity in a spared nerve injury model with a duration of action and potency that is superior to that of gabapentin [20]. Thus, σ2R activation or σ1R antagonists may promote comparable antiallodynic effects, which suggests that both types of σRs are involved in regulating neuropathic pain but have opposing effects.

Interestingly, our study suggests that σ2R is involved in the analgesic effects of MOR agonists such as morphine, DAMGO and β-endorphin. In initial experiments, no differences in baseline latencies were observed among the WT (σ2R ^+ / +^), heterozygous (σ2R ^+ / −^), and homozygous (σ2R^−/−^) groups in the warm-water tail-flick test for analgesia. Therefore, the absence of a functional σ2R did not alter thermal nociception. However, the antinociceptive effects of morphine were impaired in σ2R^−/−^ mice; the ED50 was 5 nmol in WT mice but more than 20 nmol in mice lacking σ2R. To explore the possibility that phenotypic modifications exhibited by σ2R^−/−^ mice are a consequence of compensatory mechanisms assuming the physiological functions of σ2R, we analyzed the expression of proteins implicated in the processes evaluated in our study. The mRNA expression levels of σ1R, HINT1 and MOR were similar in WT and σ2R^−/−^ mice. Most importantly, treatment with oligos to reduce the expression of σ2R mRNA diminished the responses of the mice to levels similar to those of σ2R^−/−^ mice. Because oligo treatment promotes temporary reductions in target proteins, it is unlikely that compensatory changes resulting from the absence of this protein caused the diminished response of σ2R^−/−^ mice to morphine.

Thus, our study suggest that σ2R is essential for the antinociceptive effects of exogenous and endogenous ligands of MOR but not for the antinociceptive effects of other families of G-receptors that also mediate analgesia, such as DOR, CB1R and α2AR. σ2R likely plays a relevant role in the regulation of MOR-mediated analgesia, sharing a physiological function with σ1R and glutamate *N*-methyl-d-aspartate receptor (NMDAR). The cytosolic C-terminus of MOR binds to the HINT1 protein, facilitating the interactions of σ1R and NMDAR with the MOR [22]. Notably, a lack of σ2R did not interfere with the beneficial effects of the selective σ1R antagonist S1RA on MOR-mediated analgesia. MOR agonists such as morphine increase the activity of NMDARs and then trigger a negative feedback on MOR signaling. S1RA promotes the inhibition of NMDARs by removing the σ1R from NMDAR NR1 subunits facilitating the binding of its inhibitor, calcium-activated calmodulin [22, 37]. As a result, morphine analgesia is increased and the perception of neuropathic pain is diminished [22]. As expected, this regulatory mechanism is absent in σ1R^−/−^ mice [37], but our study showed that deletion of σ2R preserved the enhancement of morphine analgesia induced by S1RA. Thus, disruption of σ1R-mediated negative control of NMDARs on MOR activity seems to account for the enhancement of the antinociceptive effects of clinically relevant opioids such as morphine, fentanyl, oxycodone, codeine, buprenorphine, and tramadol [21, 38]. Accordingly, morphine shows an enhanced capacity to produce antinociception in σ1R^−/−^ mice; 3 nmol morphine produces the same antinociceptive effect in σ1R^−/−^ mice as 10 nmol morphine does in WT mice [22]. We report here that deletion of σ1R or σ2R mostly affects MOR function but does not alter antinociception promoted by either DOR or CB1R agonists. Therefore, while σ1R inhibits and σ2R facilitates MOR-mediated analgesia these receptors exchange their roles when regulating neuropathic pain perception. Our study may open new avenues for the identification of pharmacological targets for diminishing pain perception and improving handling of opioid detoxification therapies.

## Data Availability

The data and materials of the manuscript are available upon reasonable request.

## Abbreviations

α2AR: α2-Adrenergic receptor
σ1R: Sigma-1 receptor
σ2R: Sigma-2 receptor
CB1R: Cannabinoid receptor type 1
CCI: Chronic sciatic nerve constriction injury
DAMGO: [D-Ala, *N*-MePhe, Gly-ol]-encephalin
DOR: Delta opioid receptor
DPDPE: [D-Pen2,5]-encephalin
HINT1: Histidine triad nucleotide-binding protein 1
KO (−/−): Knockout
MOR: Mu-opioid receptor
NMDAR: *N*-Methyl-d-aspartate receptor
ODN: Phosphorothioate antisense oligodeoxynucleotides
PGRMC1: Progesterone receptor membrane component 1
S1RA: 4-[2-[[5-Methyl-1-(2-naphthalenyl)-1H-pyrazol-3-yl]oxy]ethyl] morpholine
TMEM97: Transmembrane receptor 97
WT: Wild type
